# Digital smoke signals: Event-driven online search trends in Heated Tobacco Products in Poland

**DOI:** 10.18332/tpc/187280

**Published:** 2024-05-22

**Authors:** Paulina Dera, Krzysztof Klimiuk, Olga Kalinowska-Beszczyńska, Łukasz Balwicki

**Affiliations:** 1 Department of Public Health and Social Medicine, Medical University of Gdansk, Gdansk, Poland

**Keywords:** heated tobacco products, emerging products, tobacco industry marketing, public health, internet, google trends

## Abstract

**INTRODUCTION:**

Heated tobacco products (HTPs) have emerged as a prominent category in the tobacco market, particularly within European countries such as Poland. The introduction of HTPs has been met with increasing public interest, reflected in online search behavior. However, the public health implications of this new form of tobacco consumption remain a concern due to the lack of comprehensive research on its long-term effects. This study aimed to track the trends in online searches related to HTPs in Poland between 2016 and 2022, and to assess the impact of significant events on these trends in order to provide insights into public awareness of HTPs.

**METHODS:**

Utilizing Google Trends, a free source tool, this study analyzed relative search volume (RSV) for HTP-related keywords in Poland. Key events were identified and mapped against the fluctuations in RSV to understand their impact on search behavior. The analysis was confined to specific HTP-related keywords, excluding broader tobacco-related terms.

**RESULTS:**

A notable increase in HTP-related search queries was observed, particularly around the time of product launches and legislative changes. The largest increases occurred during the introduction of HTPs to the Polish market and during major events such as the COVID-19 pandemic.

**CONCLUSIONS:**

The study demonstrates a market interest in HTPs among Polish internet users, with search trends influenced by product launches and policy changes. The findings highlight the importance of monitoring online behaviors to inform public health efforts, despite limitations such as the lack of demographic data. Further research is needed to deepen the understanding of how these online trends correlate with actual consumer behavior and product sales.

## INTRODUCTION

Heated tobacco products (HTPs) are relatively new in the European tobacco market. The prototypes were announced globally in the 1980s but failed to gain popularity and were subsequently abandoned^1^. HTPs were introduced worldwide in Japan in 2014 (launch of IQOS)^2^ and in Poland in 2017^3^. Since then, their sales have been growing, particularly among the younger generation and among current and former smokers of traditional cigarettes. Recent research in Poland, conducted on a representative nationwide sample, found that 16% of current smokers had tried heated tobacco products at some point. The overall prevalence of daily use of heated tobacco in Poland was reported at 4%^4^.

HTPs work by heating tobacco at a lower temperature, which releases nicotine and other chemicals, which are then inhaled by the user. These devices pose a threat to public health due to the challenges in determining their health effects. This uncertainty is partly due to a lack of detailed toxicology research^5^. Tobacco companies exploit this limited evidence of adverse effects of heated tobacco products to craft a positive brand image. They market their products as ‘less harmful’ and involving ‘reduced risk^6^. Such marketing practices not only effectively boost sales but also influence government policies, for instance, regarding tax reliefs^7^. The current level of knowledge about HTPs is inadequate, and their increasing popularity necessitates the collection and analysis of more data^8^.

Over the years, the significance of online marketing for the tobacco industry, and specifically the use of social media marketing to attract new customers, have increased. These have become fundamental tools for brand management.

Social media marketing has evolved into an effective means for social engagement, functioning not only as a platform for product promotion but also for strengthening brand image. Compared to traditional methods, it enables targeting of marketing efforts toward specific demographic sectors through the use of sophisticated tools such as statistical analyses, trend monitoring, and adaptation of popular information technologies, such as keywords and hashtags^9-11^. According to data from January 2022, over 80% of all Polish citizens use the internet. This number is on the rise. In 2022, the proportion of social media users increased by 5% compared to the previous year, with about 72% of the total population engaging in social media^12^. It has been observed that social media users exposed to content promoting tobacco products or tobacco industry companies, face a higher risk of tobacco addiction compared to those who are not exposed^13,14^. Given these data, regulating content in social media marketing^15^ is crucial for mitigating its negative impact on society. Moreover, from a public health standpoint, this connection underscores the utility of analyzing search query data, which provides insights into current behaviors and can potentially forecast future trends^16^.

Poland is recognized as one of the most important markets for emerging products such as HTPs, considering the prevalence of their use and intensive marketing. That is why public health experts and government bodies require insight into the pro-smoking activities conducted by tobacco companies. Equipped with these data, as well as knowledge of how the market is functioning, they can devise preventive measures and health promotion initiatives. Regrettably, there is a dearth of research on social media marketing activities of the tobacco industry in Poland, therefore creating a gap in knowledge that needs to be addressed^17-19^.

Google is the most widely used search engine. In Poland, nearly 96% of internet searches are conducted through Google^20^. Google Trends analysis offers an opportunity to utilize search data to study the current popularity of HTPs online, particularly because it is free and publicly available, while other data are not easily accessible. Researchers are increasingly adopting new tools, among which Google Trends is prominent. Its use in medical and public health research is growing^21-23^. Given this trend, it was determined that combining data from Google Trends with key events would serve as an appropriate methodology for this research.

The primary aim of the study was to identify trends in searches for keywords related to heated tobacco products (HTPs) across the Polish internet between 2016 and 2022. A secondary objective was to analyze how public awareness of and interest in HTPs, as reflected in these search queries, are influenced by various external factors such as legislative changes, health campaigns, and marketing strategies of tobacco companies. This involved examining co-occurrences of significant national and international events and the fluctuating search interest in HTP-related terms, thereby providing insights into consumer behavior and the effectiveness of public health strategies.

## METHODS

### Study design and procedure

The study used Google Trends (GT). GT is a free source tool and provides information on the relative popularity of search queries over time (relative search volume, RSV) for a specific location and time period. These data are free, publicly available, and regularly updated. RSV is an index adjusted to the number of Google searches in a given geographical area, ranging from 1 to 100, where 100 indicates the highest popularity in the given period and location, and 0 represents the lowest level (<1%)^21^. Google Trends compiles and processes data from a number of sources, including Google Search, Images, News, Shopping, and YouTube^24^.

Additionally, key events occurring in Poland or globally in the given period were observed. These events were categorized as ‘legislation, ‘marketing’, or ‘health campaigns’ (Table 1). Event selection was informed by a review of literature, legal Acts, marketing activities of tobacco industry companies, and anti-tobacco campaigns, which were agreed upon by the authors of this research. The RSV data for individual keywords were then compared with events occurring during the selected time to identify potential relationships. However, it was not possible to ascertain the motivations behind the searches for any keywords.

**Table 1:** Selected key events included in the analysis, Gdansk, Poland, 2023

*No.*	*Key event*	*Time of occurrence*	*Type of event*
1	Regulation of e-cigarettes by Act of 22 July 2016 amending the Act on protection of health against the consequences of using tobacco and tobacco products	September 2016	National law regulation
2	Worldwide launch of GLO	November 2016	International HTP marketing
3	PMI submission of the application to FDA to classify IQOS as a ‘modified risk tobacco product’	December 2016	International law regulation
4	Launch of IQOS in Poland	April 2017	National HTP marketing
5	FDA recognizes that PMI data show reduced exposure to harmful substances but disagrees that it reduces the risk of illness and death, and does not recognize this product as a product of the ‘risk modification’ standard	January 2018	International law regulation
6	Polish nationwide anti-tobacco campaign ‘Niespalsienastarcie’	January 2018	National public health campaign
7	Launch of GLO in Poland	March 2018	National HTP marketing
8	Consultations on the draft amending the Act on excise duty and certain other Acts in Poland	December 2019 – January 2020	National law regulation
9	Polish nationwide anti-tobacco campaign #STOPFEJKFRIENDS	January 2020	National public health campaign
10	COVID-19 epidemic in Poland	March 2020	National health status
11	Ban on the sale of flavored cigarettes in Poland	May 2020	National law regulation
12	Publication of report suggesting that nicotine may be a novel potential Cytokine Release Syndrome (CRS) therapy in severe COVID-19 patients	June 2020	International health status
13	Delaying the introduction of the excise tax for e-cigarette liquids in Poland	July 2020	National law regulation
14	Polish presidential election	July 2020	National authority election
15	The US FDA authorization of the marketing of a heated tobacco product, the IQOS Tobacco Heating System, under the Federal Food, Drug and Cosmetic Act	July 2020	International law regulation
16	GLO advertising campaign starts in Poland	January 2021	National HTP marketing
17	Act of 1995: Protection of health against the consequences of the use of tobacco and tobacco products – Change in Article 5. It is forbidden to smoke tobacco products, including smoking innovative tobacco products, and to smoke electronic cigarettes	January 2021	National law regulation
18	Increase in excise duty on cigarettes	January 2022	National law regulation

Search data for the analyzed queries in the Polish Google data sources from January 2016 to December 2022 were exported on 30 January 2023, using Google Trends. The study dates were chosen based on the market launch of IQOS in April 2017 and GLO in March 2018 in Poland. These brands were chosen because they are the main HTP products from the two most popular tobacco companies in Poland, Philip Morris and British American Tobacco (BAT)^25^. It was important to observe the popularity of selected keywords both before and after these products’ market launch, as companies typically intensify their communication strategies beforehand.

The analysis of the imported data was conducted using Microsoft Excel (Office 365 version). Key events were compared with an RSV value, and charts were created only for ‘IQOS’ and ‘GLO’ to better visualize changes over time. These two keywords were chosen due to the high popularity of particular HTP devices. The remaining figures only show changes in RSV values for the other keywords.

### Data collection

The study focused on two best-selling heated tobacco products in the heated tobacco market along with related keywords, as shown in Table 2.

**Table 2:** Selected keywords with a description used for Google Trends data retrieval, Gdansk, Poland, 2023

*Keyword*	*Description*
IQOS	Philip Morris International (PMI) heated tobacco product
GLO	British American Tobacco (BAT) heated tobacco product
‘Podgrzewacz tytoniu’	Polish for ‘tobacco heater’
HEETS	Tobacco sticks for IQOS
FIIT	Tobacco sticks for lil SOLID 2.0 (PMI’s heated tobacco product), also compatible with IQOS
‘Wkłady tytoniowe’	Polish for tobacco sticks
‘Wkłady do IQOS’	Polish for sticks for IQOS
‘Wkłady do GLO’	Polish for sticks for GLO

Exclusion criteria covered other keywords associated with tobacco products, such as traditional cigarettes and e-cigarettes. The term ‘tytoń podgrzewany’ (Polish for heated tobacco) was also excluded due to a low number of searches. The heated tobacco product ‘NEO’ was not included because of the multiple meanings of the word ‘neo’. Keywords were grouped into several categories: names of heated tobacco products from major tobacco companies in Poland, names of tobacco sticks for selected products, and phrases related to the above.

## RESULTS

**Figure 1:**
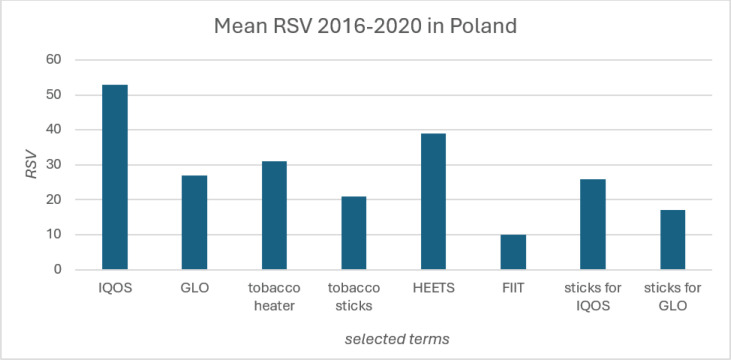
Comparison of mean RSV of selected terms, Gdansk, Poland, 2023 (N=224)

**Figure 2:**
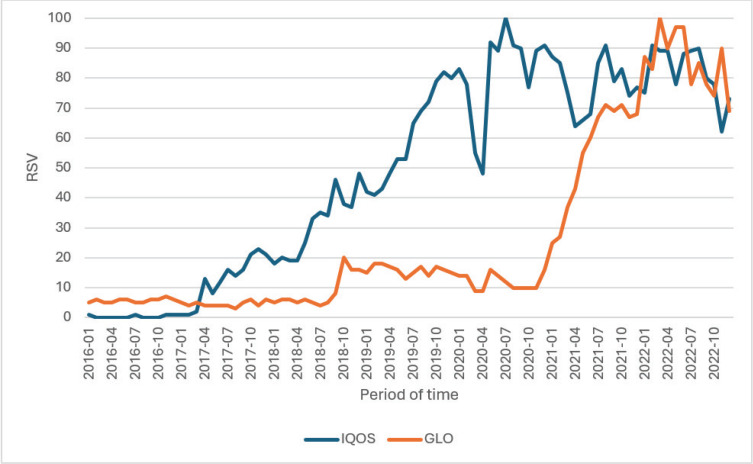
Comparison of relative search values for the most popular brands of heated tobacco products, Gdansk, Poland, 2023 (N=6397)

The popularity of heated tobacco products in Poland has seen a significant increase in recent years, particularly between 2018 and 2021^26^. Tobacco products from companies with the largest market share have had the most substantial impact on search trends in Poland. An analysis of the keywords ‘IQOS’ and ‘GLO’ revealed that the mean RSV was higher for the former (53 vs 27) (Figure 1). However, initial searches in 2016 were more frequent for the second word, as shown in Figure 2. Additionally, the overall mean RSV for selected keywords demonstrates the dominance of Philip Morris products (‘IQOS’, ‘HEETS’, ‘Wkłady do IQOS’) over others.

**Figure 3:**
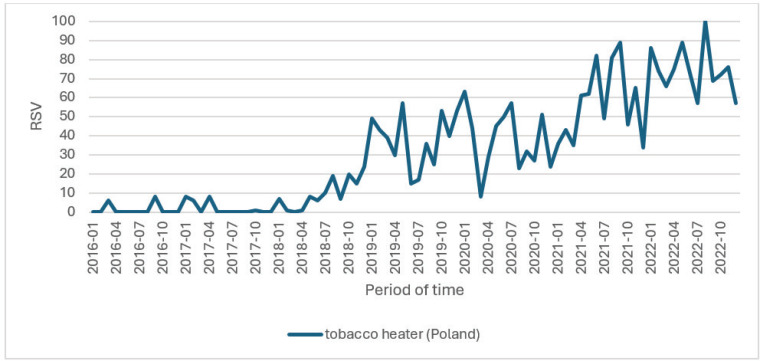
Queries on Google for ‘podgrzewacz tytoniu’ (tobacco heater), Gdansk, Poland, 2023 (N=2642)

The interest in the device itself, referred to as a ‘tobacco heater’, has shown a fluctuating increase, as depicted in Figure 3.

**Figure 4:**
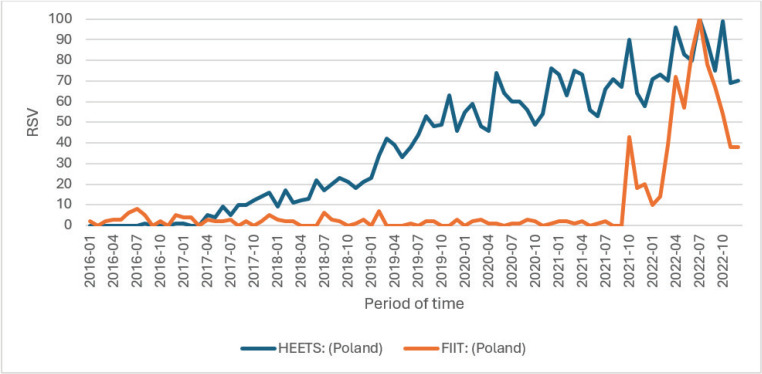
Comparison of relative search values for the most popular brands of tobacco sticks, Gdansk, Poland, 2023 (N=4146)

Data on specific brands of tobacco sticks (Figure 4) indicated that searches for ‘HEETS’ have been consistently increasing since 2017. In contrast, ‘FIIT’ was introduced to the Polish market in December 2021^27^, leading to a gradual increase in queries for this term since its launch. As indicated in Table 2, FIIT is compatible with the same device (IQOS) as HEETS, allowing the users of heated tobacco products to choose between the two brands.

**Figure 5:**
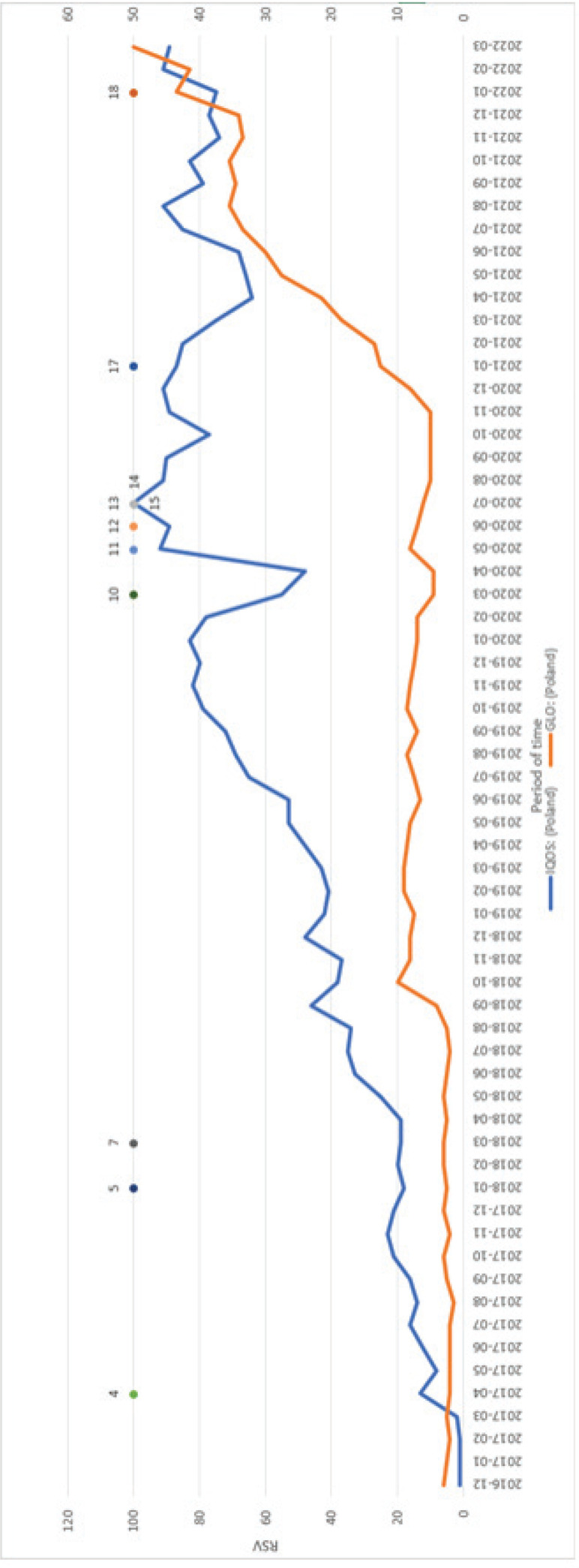
Comparison of relative search value for the most popular brands of heated tobacco products with selected key events as listed in Table 2, Gdansk, Poland, 2023 (N=6369)

The analysis of key events in relation to selected terms for the most popular heated tobacco products in Poland revealed some possible correlations with their popularity (Figure 5). The first surge in searches for ‘IQOS’ coincided with its launch in April 2017. Conversely, the observation results showed a slight decline in the RSV for both ‘IQOS’ and ‘GLO’ in January 2018, following the FDA’s acknowledgment of PMI data showing reduced exposure to harmful substances and the Polish nationwide anti-tobacco campaign ‘Niespalsienastarcie’. The popularity of ‘IQOS’ and ‘GLO’ rebounded in March 2018 with the launch of GLO in Poland. The most significant relative decrease in search volume for the selected terms occurred concurrently with the announcement of the COVID-19 epidemic in Poland in March 2020. Another notable increase was observed in May 2020, coinciding with the ban on the sale of flavored cigarettes in Poland. Additionally, the highest RSV for ‘IQOS’ was recorded in July 2020, during the delay in the introduction of the excise tax for e-cigarette liquids, the presidential election, and the US FDA’s authorization of marketing HTPs. FDA decisions have an impact not only in the US but also in other countries. Due to the decisions issued by this organization, a discussion about the validity of the decision began in the Polish media. Finally, following the increase in excise duty on cigarettes, searches for ‘IQOS’ and ‘GLO’ surged.

## DISCUSSION

The findings of our study represent a significant breakthrough in tobacco research in Poland, revealing a marked increase in online searches concerning emerging tobacco products closely linked with key events like product launches, the COVID-19 pandemic, and legislative changes. This pioneering research in Poland, the first to comprehensively combine Google Trends data with the timeline of major events, provides a novel perspective that transcends the scope of existing reports like the Tobacco Industry Interference Index^28^. By adding a societal dimension to the analysis of search behavior and its interaction with industry and government actions, our study not only advances scientific understanding but also underscores the critical need for ongoing surveillance of how these entities, as indicated earlier, influence public perceptions and behaviors around tobacco use.

A noteworthy strength of our research is the comprehensive analysis of search trends over a six-year period, highlighting the evolving public interest in heated tobacco products in Poland. Especially if we look at the dates between 2018 and 2022, where other studies have shown a significant increase in HTP popularity, this longitudinal approach provides valuable insights into the changing dynamics of interest in tobacco products in response to various external influences.

Comparing our results with other studies, we find similarities in the influence of government actions and public health events on public interest in tobacco-related topics. For instance, the 2009 US cigarette excise tax increase significantly impacted US-wide interest in quitting smoking^22^. Our study similarly indicates that tax changes and legislative actions in Poland influenced search trends for heated tobacco products.

The launch of heated tobacco products like ‘IQOS’ and ‘GLO’ in Poland led to a noticeable increase in related search queries, mirroring trends observed in Japan following the introduction of IQOS^23^. This parallel underscores the global influence of new tobacco product launches on public interest and the importance of monitoring these trends for public health and policy planning.

In addition to Google Search, Google Shopping also serves as a data source for Google Trends, allowing for the observation of behavioral shifts in purchases of HTPs during health crises such as the EVALI outbreak. At the height of this outbreak, there was a pronounced spike in IQOS-related searches, reflecting the public perception of IQOS as a safer alternative to vaping^29^. However, in contrast to these findings, the Polish internet showed a rapid decline in searches for ‘IQOS’ and ‘GLO’, but in a different period.

Our inability to measure the motivation behind searches about these products does not preclude the observation of HTP perception on social media. On Twitter, for example, users generally displayed a more negative or neutral stance toward IQOS rather than a positive one. This sentiment aligns with the behavioral changes following the FDA’s enforcement policy on unauthorized flavored e-cigarettes, which seemingly impacted public attitudes toward new tobacco products^30^. This sentiment aligns with the behavioral changes following the FDA’s enforcement policy on unauthorized flavored e-cigarettes, which seemingly impacted US public attitudes toward new tobacco products. Moreover, the discourse on Twitter was influenced by regulatory events, such as the initial rejection and subsequent permission to sell IQOS in 2019, which stimulated a surge in tweets from opposing e-cigarette advocacy groups^31^. Instagram’s influence on public perception of new nicotine and tobacco products is also noteworthy. A significant portion of #IQOS hashtag users consists of official brand accounts, retailers, and influencers promoting these products, underscoring the role of social media in shaping consumer interests^32^.

The relationship between product popularity and online searches is evident. Studies from the US indicate a positive correlation between Google search volumes for non-cigarette tobacco products and the prevalence of their use among youth and adults, reflecting how online interest can mirror real-world consumption trends^33^.

The pervasive impact of online information on daily life, highlights the importance of monitoring and analyzing digital content. While current studies may not fully reflect the latest trends, they provide a template for future research^34-36^. Further investigations, particularly those comparing Google Trends data with actual sales figures, could yield additional insights into the effectiveness of marketing strategies and regulatory policies.

### Limitations

The study’s limitations are important to consider for a nuanced interpretation of the results. First, due to data anonymization in Google Trends, we lacked specific demographic information such as the age or gender of the individuals conducting the searches, only obtaining the geopolitical region of the search. This restriction potentially limits the understanding of the diverse search behaviors across different social groups within Poland. Secondly, the study focused on individuals with internet access and those using Google Search, which may not accurately represent the entire Polish population’s interest in heated tobacco products.

Additionally, the keyword selection was narrowed to exclude other tobacco products like traditional cigarettes and e-cigarettes. Terms like ‘tytoń podgrzewany’ (Polish for heated tobacco) and ‘NEO’ were not considered due to their low data volume or multiple meanings. This selective approach could have affected the study’s comprehensiveness. Moreover, the research did not consider specific search phrases related to purchasing locations, such as ‘where to buy IQOS’, potentially leading to an incomplete picture of consumer interest and behavior.

Lastly, the selection of key events chosen as having a possible influence on search volumes might have been biased or inaccurate, affecting the study’s conclusions on the relationships between these events and search trends in Poland.

## CONCLUSIONS

Our study provides critical insight into the online search patterns related to heated tobacco product use in Poland. It highlights how significant events, such as product launches and policy changes, can influence public interest in alternative tobacco products. The co-occurrence identified between online search behavior and these events underscores the value of internet search data as a tool for public health surveillance and policy-making. Despite the limitations of the study, including anonymized demographic data and a focus on specific keywords, the findings underscore the importance of continuous monitoring of the digital landscape to better understand public health trends. Future research should aim to integrate the information on these online trends with actual sales data to enhance the understanding of consumer behavior toward tobacco products.

## Supplementary Material



## Data Availability

The data presented in this study are available on request from the corresponding author.
