# Supplementary material for: Digital smoke signals: Event-driven online search trends in Heated Tobacco Products in Poland
Source: Tob Prev Cessat. 2024 May 22;22:10.18332/tpc/187280. doi: 10.18332/tpc/187280 (PMC11112517; doi:10.18332/tpc/187280)
Supplement: Supplementary file 1 [file TPC-10-22-s1.pdf]

Supplementary Material

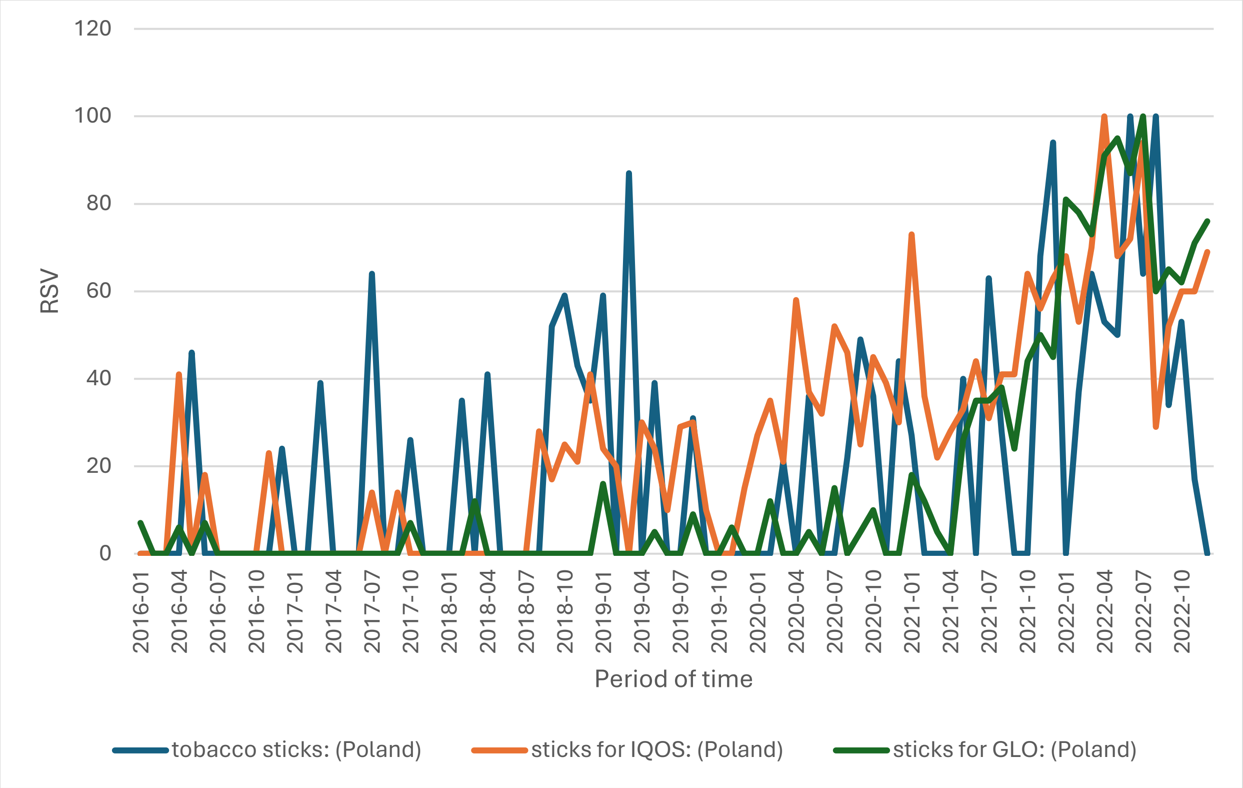

Figure 6. Comparison of RSV values for Google searches for the "tobacco sticks" related terms; 2023; Gdansk, Poland; n=5381 (sample size)

© 2024 Dera P. et al.
